# Improvement of the PLA Crystallinity and Heat Distortion Temperature Optimizing the Content of Nucleating Agents and the Injection Molding Cycle Time

**DOI:** 10.3390/polym14050977

**Published:** 2022-02-28

**Authors:** Laura Aliotta, Letizia Maria Sciara, Patrizia Cinelli, Ilaria Canesi, Andrea Lazzeri

**Affiliations:** 1 Department of Civil and Industrial Engineering, University of Pisa, 56122 Pisa, Italy; patrizia.cinelli@unipi.it; 2 Interuniversity National Consortium of Materials Science and Technology (INSTM), 50121 Florence, Italy; 3 Planet Bioplastics s.r.l., 56127 Pisa, Italy; letizia.m.sciara@gmail.com (L.M.S.); ilariacanesi@planetbioplastics.com (I.C.)

**Keywords:** poly (lactic acid), injection molding, heat deflection temperature, impact strength, nucleating agent

## Abstract

Three different commercial nucleating agents (LAK, talc, and calcium carbonate) were added at different weight percentages into poly (lactic acid) (PLA) in order to investigate the mechanical and thermo-mechanical behavior of blends in correlation to injection molding parameters. After as-sessing the best content of each nucleating agent, analyzing isothermal and non-isothermal crys-tallization, two cycle times that can be industrially adopted were selected. Crystallinity highly impacts the flexural modulus, while it improves the heat deflection temperature only when the crystallinity percentage is above 50%; nevertheless, an excessive crystallinity content leads to a decrement of impact resistance. LAK does not appear to be sensitive to cycle time while talc and calcium carbonate proved to be effective if a cycle time of 60 s is adopted. Since the choice of nu-cleating agent is not univocal, the identification of the best nucleating agents is subject to the technical specifications required by the application, accotuing for the most important commercial requirements (productivity, temperature, and impact resistance).

## 1. Introduction

Due to the environmental pollution resulting from the use and disposal of non-degradable fossil-based polymers, replacement of fossil-based polymers with biodegradable renewable biopolymers has received great attention in recent years. Numerous research activities carried out both by researchers and industries have contributed to the wide availability of biopolymers that are on the market today. The use of biopolymers contributes to the reduction of the environmental impact generated by fossil-based plastics, especially in sectors such as packaging, textiles, automotive manufacturing, additive manufacturing, etc. [1,2,3,4,5,6].

Its renewability, compostability, and good mechanical properties make PLA a very promising biopolymer. The large-scale production of high-molecular-weight PLA has broadened its uses [7]. However, PLA application is still limited by its fragility and low thermal stability. Its glass transition temperature (about 60 °C), low heat deflection temperature (HDT), and slow crystallization rate severely limit PLA application in those sectors where temperature resistance is required [8,9].

The low HDT drawback of PLA can be overcome by PLA crystallization due to the capacity of the crystalline PLA phase to achieve high HDT improvements [10,11]. Moreover, the mechanical properties of PLA are strictly dependent upon the crystallinity content and morphology of the crystalline structure [12], and their control and modulation is fundamental to obtaining tailored thermo-mechanical properties according to the final use of the material.

The control of the PLA crystallization temperature is an important parameter to be designed; during the processing condition, in fact, the variation of the PLA crystallization temperature can encourage the formation of one PLA crystalline structure rather than another [13,14]. Nevertheless, under normal industrial processing conditions (such as extrusion and injection molding) the α-form is commonly generated; this crystalline form grows during melt and cold crystallizations at temperatures higher than 110 °C. At temperatures lower than about 100 °C, the disordered α′-form was observed [15,16,17,18,19] and it was obtained during the processing, adopting particular injection molding parameters [12].

Injection molding is the most widely used process for manufacturing a variety of parts that can have complex shapes, require high dimensional precision, and at the same time that must be produced in large amounts in a short time [20]. The importance of setting the correct molding conditions plays an important role not only because they affect the quality of the molded product (presence of defects, warpage, shrinkages, or residual stress) but also because they can affect the productivity, cycle time, and energy consumption of the process [21].

PLA crystallization during injection molding is fundamental to obtaining pieces with improved HDT, stiffness, and chemical resistance [22]; however, it must be considered that a decrease in impact strength could be induced by an increasing crystallinity content [23].

From an industrial point of view, the major PLA drawback for the injection molding applications is the slow PLA crystallization time that is reflected in longer molding cycles when compared to conventional fossil-based polymers. Furthermore, the molding temperature also complicates the PLA injection molding process. In fact, while for most of the fossil based polymers the material can be injected into molds at room temperature, for PLA it is not possible because the resulting material proves to be amorphous with low storage modulus, especially at temperatures above its glass transition temperature [22,24].

Consequently, PLA needs to be crystallized under injection molding conditions; this can be achieved by increasing both the mold temperature and the cycle time. Nevertheless, in order to make the process feasible from an industrial point of view, minimizing the extra costs generated by the use of hot molds and increased cycle time, the crystallization rate of PLA must be accelerated during the process.

To this purpose, different approaches (such as block copolymerization, chemical modification, nucleation, plasticization, and physical blending with other polymers) have been investigated to achieve high PLA crystallinity in a short time [11,23,25,26,27,28,29,30]. Among the mentioned approaches, the nucleation approach is very attractive because it can shorten the injection molding time while not significantly affecting the PLA strength and rigidity. Furthermore, the nucleation approach is very versatile (the nucleating agents are easily added during extrusion [31]) and up-to-date different nucleating agents (NAs) can easily be found on the market.

In the literature, many potential PLA nucleating agents have been reported to be effective in increasing not only PLA crystallinity content but also in improving mechanical, optical, and heat resistance and the processability of PLA. The addition of NAs reduces the surface free energy barrier towards nucleation leading PLA to crystallize at higher temperatures [32]. In particular, good results were achieved with the use of: PDLA, LAK (an aromatic sulphonate derivative), boron nitrate, talc, calcium carbonate, natural fibers, and zinc phenylphoshonate [14,33,34,35]. It has been observed by Schafer et al. [36] that the reduction in cycle times using biobased nucleating agents is capable of adopting elevated mold temperatures or carrying out a post-heat treatment of injection molded specimens. Some encouraging results were achieved using orotic acid (OA), N’1,N’6-dibenzoyladipohydrazide (TMC-306) and N1,N1-(ethane-1,2-diyl)bis(N2-phenyloxalamide) (OXA), and titanium dioxide (TiO_2_) as nucleating agents for PLA; however, their application in competitive injection molding cycle times has not been explored yet [32,37,38].

New typologies of nucleating agents for PLA have been also investigated recently (such as zinc salts of amino acids [39], CdSe/ZnS quantum dots [40], and carbon nanotubes [41]), but they are still far away from being commercialized at competitive costs.

The optimization of injection molding conditions with commercial and cost-competitive nucleating agents is thus fundamental. In the present study, three different traditional NAs were investigated (LAK, calcium carbonate, and talc), and they were added into PLA in varying amounts with the aim of determining the best concentration for direct crystallization during the injection molding process, adopting injection cycle times that can be adequate for industrial production.

In this study, particular attention was paid to the choice of the mold temperature and injection cycle time. These two parameters are crucial and interconnected. A high mold temperature is required (otherwise the PLA crystallization rate is too slow with NA addition) that, if coupled with low cycle time, can induce deformation when the PLA sample is extracted from the mold [11]. The cycle times were selected to be as low as possible (30 s and 60 s) with a mold temperature of 110 °C that has been reported to be the minimum temperature to be effective for efficiently crystallizing PLA [12,14]. For the cycle time, 60 s was exceeded because, from an industrial processing point of view, cycle times exceeding 60 s are not preferred if an acceptable final material cost has to be maintained [10].

Isothermal and non-isothermal crystallization, and self-nucleation experiments—including the final crystallinity content of the injection molded PLA sample—were investigated by differential scanning calorimetry (DSC). The mechanical performance (flexural and elastic modulus, tensile strength, impact resistance, and HDT) were also evaluated and correlated to the final crystallinity achieved. The selection of the suitable quantity and NA to be added to tune the final PLA properties—balancing the thermal, mechanical, and injection molding cycle time—was thus carried out. Interesting results were also achieved in terms of both crystallinity and mechanical property improvements. In fact, it has been observed that not all the NAs used can guarantee prevention of the decay of the Charpy impact strength and contemporary HDT improvement.

## 2. Materials and Methods

### 2.1. Materials

The materials used in this work were:

- Poly(lactic) acid (PLA), trade name PLA 3100 HP, purchased from Natureworks LLC (Minneapolis, MN, USA). It is commercial-grade PLA (~0.3% of D content) derived from natural resources and designed for injection molding applications (density: 1.24 g/cm^3^; melt flow index (MFI) (210 °C/2.16 kg): 24 g/10 min, Mw = 148,250 g/mol).
- Potassium salt of 3,5-bis(methoxycarbonyl)benzenesulfonate, trade name LAK-301, produced by Takemoto Oil & Fat (Minatomachi, Japan), is an aromatic sulphonate derivative nucleating agent. It appears as a whitish powder, with a specific gravity 1.668 g/cm^3^ and particle size around 10 μm.
- Jetfine^®^ 0.7 CA talc provided by IMERYS (Paris, France). It is an ultrafine-grind lamellar talc able to improve the nucleation in crystalline polymers. It appears as a very white powder, with specific gravity of 2.78 g/cm^3^ and medium particle size of 0.7 μm.
- Socal^®^ 312 calcium carbonate was also provided by IMERYS (Paris, France). It is an ultrafine, white and odorless, organic, surface coated and precipitated calcium carbonate. It is a powder with unique crystal size and shape (density: 2.71 g/cm^3^; particle diameter: 0.05–0.09 μm; surface area: 18 m^2^/g; coating content: 24–33 g/kg).

### 2.2. PLA Formulation Extrusion and Injection Molding

PLA pellets were dried in a Piovan DP 604-615 dryer (Piovan S.p.A., Verona, Italy) at 60 °C for 8 h before processing. The three different NAs were mixed to PLA according to the compositions reported in Table 1. The blends were prepared using a semi-industrial twin-screw extruder Comac EBC 25HT (L/D = 44) (Comac S.r.l., Cerro Maggiore, Italy). PLA granules were fed through the main hopper while the NAs were added by a lateral suitable feeder, calibrated for feeding the correct weight amount of NAs during the extrusion. The temperature profile of the 11 extruder zones was: 150/175/185/190/190/185/185/185/180/180/180 while the total mass flow rate was set at 18 kg/h at 300 rpm.

The strands coming from the extruder dies were cooled in a water bath at room temperature and shaped into pellets by an automatic knife cutter. The pellets were then finally dried at 30 °C for 8 h in a PIOVAN dryer.

The injection molding was carried out on a Megatech H10/18–1 (TECNICA DUEBI S.r.l, Fabriano, Italy) that could obtain specimens according to ISO 527-1A (with dimensions of 80 × 10 × 4 mm). The injection molding parameters adopted are summarized in Table 2. In order to obtain injection molded specimens with conditions as close as possible to each other, the same injection temperature profile for all formulations was adopted. The molding temperature was set at 110 °C and two different cycle times (the sum of injection, holding time, and cooling time) were adopted: 30 s and 60 s.

### 2.3. Thermal Characterizations

Differential scanning calorimetry (DSC) measurements were carried out with a TA Q200 (TA Instruments, New Castle, UK) equipped with a RSC90 cooling system. The instrument was calibrated with indium as standard, using aluminium hermetic pans and nitrogen as purge gas set at a rate of 50 mL/min. Thanks to the preliminary thermal investigation, the best concentrations of the various different nucleating agents were selected for further mechanical characterizations.

#### 2.3.1. Non-Isothermal Crystallization

For non-isothermal melt crystallization, the samples to be analyzed have been chosen from the extruded pellets that were rapidly heated at 60 °C/min to 190 °C and held for 3 min to erase the thermal history, then the melt was cooled to crystallize at a rate of 5 °C/min in order to evaluate the cold crystallization temperature (*T_c_*).

#### 2.3.2. Isothermal Crystallization

Isothermal melt crystallization kinetics of neat PLA and its formulations were also investigated using the Avrami equation. To this purpose, the following thermal program was carried out on the extruded pellets: the thermal history of each sample was erased by a ramp of 60 °C/min to 190 °C, followed by an isothermal step of 3 min; then, a fast cooling at 100 °C/min to the desired crystallization temperature *T_c_* was carried out and the sample was held at the chosen crystallization temperature until the isothermal crystallization was completed. The crystallization temperature for the isothermal analysis was chosen equal to the mold temperature (110 °C) adopted during the injection molding process.

The isothermal melt crystallization kinetics were determined adopting the well-known Avrami equation [42,43] in which it is assumed that the relative crystallinity, *X(t),* changes with the crystallization time, *t*, according to Equation (1),

(1) $$1-X\left(t\right)=\exp\left(-kt^{n}\right)$$

where *n* is the Avrami exponent and *k* is the crystallization rate constant which involves both nucleation and rate growth parameters. To better compare the effectiveness of the NAs addition on the PLA crystallization kinetic, the crystallization half-time (*t*_0.5_) was calculated as [44]

(2) $$t_{0.5}={\left(\frac{ln2}{k}\right)}^{1/n}\;\;\;$$

where *t*_0.5_ is defined as the time needed to achieve 50% of the final crystallinity of the samples.

#### 2.3.3. Final Thermal Properties and Crystallinity of the Injection Molded Specimens

To evaluate the final crystallinity reached from the materials after their injection molding, about 10–15 mg of material were cut from the molded samples. The sampling was carried out exactly in the same region of the injection molded specimens. Only the first run was considered and the thermal program adopted was: heating at 10 °C/min from room temperature to 190 °C, followed by an isothermal step of 1 min.

The PLA melting temperature (*T_m_*) and the cold crystallization temperature (*T_cc_*) were measured in correspondence of the maximum of the melting peak and of the minimum of the cold crystallization peak, respectively. The enthalpies of melting (*ΔH_m_*) and cold crystallization (*ΔH_cc_*) were determined from the corresponding peak areas in the heating thermograms.

The PLA crystallinity percentage (*X_cc_*) was calculated as

(3) $$X_{cc}=\frac{\Delta H_{m}-\Delta H_{cc}}{H{{}^{\circ}}_{m}\cdot \left(1-wt.\%\;of\;nucleating\;agent\right)}\;\;\;$$

The theoretical melting of 100% crystalline PLA was taken equal to 93 J/g [45].

#### 2.3.4. Self-Nucleation and Nucleating Efficiency Evaluation

**Figure 1:**
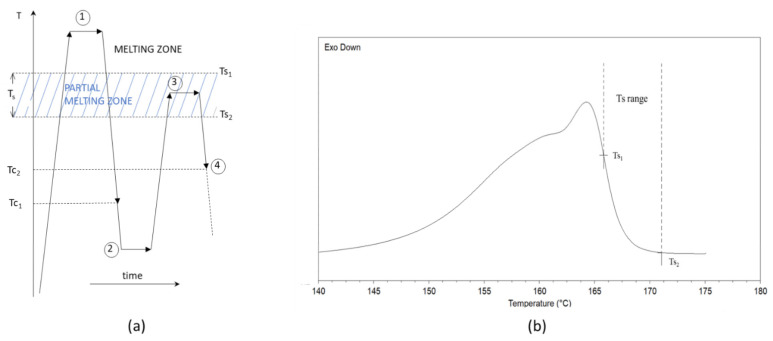
(**a**) Four-step DSC method for self-nucleation procedure; (**b**) Melting endotherm of PLA in the “standard” state (i.e., after crystallization at T_cl_) and indication of the partial melting range used in self-nucleation experiments (T_S_).

In order to evaluate the nucleation efficiency of the various NAs, a self-nucleation experiment was also carried out on neat PLA extruded pellets. The adopted experimental procedure followed the work of Wittmann et al. [46,47,48] in which the procedure for the construction of a calorimetric nucleation efficiency scale for PLA can be found. The procedure adopted can be divided into a four-step DSC method that is necessary to control the self-nucleation procedure (Figure 1a):

1. Erasing of the sample thermal history: in this first step, PLA was rapidly heated at 200 °C/min to 210 °C and held at this temperature for 5 min to erase its thermal history.
2. Creation of the standard state (*T*_*c*1_): this standard state is obtained by cooling the sample from point 1 at 10 °C/min to a temperature below its cold crystallization temperature. For this step, a temperature equal to 65 °C was chosen. During this step, the crystallization takes place at the lower limit of the crystallization range (*T*_*c*1_) depending on the molecular polymer characteristics.
3. Partial melting self-nucleation: this is the fundamental step for self-nucleation and it was obtained by heating the sample at 10 °C/min to selected temperatures, *T_s_*, ranging from *T*_*s*1_ = 164 °C to *T*_*s*2_ = 171 °C; then it followed an isothermal step of 5 min. The *T_s_* is located in the temperature range illustrated in Figure 1b where the formation of stabilized polymer crystal fragments occurred. The concentration of the crystal fragments varies in the *T*_*s*1_–*T*_*s*2_ range and it increases as *T_s_* decreases reaching the saturation for *T*_s_ = *T*_*s*2_.
4. Final crystallization (*T*_*c*2_): in this last step, a second crystallization is achieved by cooling the sample by 10 °C/min to 65 °C. At this point, the crystallization peak will be located at *T*_*c*2_ (with *T*_*c*2_ ≥ *T*_*c*1_). This *T*_c_ increment is correlated to an increment of the nucleation site concentration induced by the self-nucleation process. Consequently, the PLA sample (not self-nucleated) crystallizes at the lowest temperature, *T*_*c*1_, whereas the best self-nucleated samples crystallizes at the highest temperature, *T*_*c*2*,max*_.

The addition of a NA improves the crystallization, bringing to *T_c_* higher than *T*_*c*1_; consequently, the nucleation efficiency (*NE*) can be calculated as [46,48]

(4) $$NE=\;\frac{T_{c}-T_{c1}}{T_{c2,max}-T_{c1}}\cdot 100\;\;\;$$

where *T*_*c*2*,max*_ and *T*_*c*1_ are calculated for neat PLA according to the four-step procedure above mentioned; *T_c_* is the crystallization temperature recorded for PLA containing the NA. If no nucleating action is registered, NE will be equal to 0.

### 2.4. Mechanical Characterization

The mechanical characterizations were performed after 2 days from the injection molding process; during this time, the specimens were stored in a dry keeper (SANPLATEC Corp., Osaka, Japan) at controlled temperature and humidity (25 °C and 50% r. h).

The tensile tests were carried out at room temperature with an MTS Criterion 43 universal testing machine (MTS Systems Corporation, Eden Prairie, MN, USA) interfaced with an MTS Elite Software (MTS Testsuite version 4.1). The machine, equipped with a 10 kN load cell, was set at a constant crosshead speed of 10 mm/min. At least 10 specimens were tested, and the average values of the main mechanical properties were reported.

The flexural modulus was evaluated using the above-mentioned MTS machine in a three-point bending (3PB) configuration with the specimen in a flatwise position. The crosshead was set at 2 mm/min, and the sample dimensions for the 3PB tests were: 80 × 10 × 4 mm. At least five samples for each formulation were tested and the mean flexural modulus value was reported. Flexural modulus (*E_B_*) was calculated from stress–strain curves according to ASTM D790 using the following equation

(5) $$E_{B}=\;\frac{L^{3}m}{4bd^{3}}$$

where *L* is the support span; *b* and *d* are the width and the thickness of the sample tested, respectively; and *m* is the angular coefficient of the linear elastic part of the load–deflection curve (N/mm).

The impact tests were performed using V-notched ISO 179 parallelepiped specimens with Instron CEAST 9050 machine (INSTRON, Canton, MA, USA). At least 10 specimens for each blend were tested at room temperature.

### 2.5. Heat Defection Temperature (HDT) Measurements

The heat deflection temperature or heat distortion temperature (HDT) is defined as the temperature at which a polymeric material undergoes to deformation under a specified load. This property is fundamental during the design and production of thermoplastic components, and it is strictly correlated to the polymer crystallinity. Generally, a highly crystalline polymer has an HDT value higher than its amorphous counterpart [49,50]. The determination of HDT was carried out on a CEAST HV 3 (INSTRON, Canton, MA, USA) in accordance with ISO 75-1 (method A). The sample, a parallelepiped with dimensions of 80 × 10 × 4 mm, is immersed in a silicone oil bath and subjected to a flexural stress of 0.45 MPa at the midpoint of the flatwise position of a 3PB configuration. The test starts with heating the bath at a heating rate of 120 °C/h. When the sample bar reaches a deflection of 0.34 mm, the corresponding bath temperature represents the HDT (Type A) value. At least five measurements were carried out and the average value is reported here.

## 3. Results and Discussion

### 3.1. Identification of the Best Nucleating Agent Content

The injection molding process can be seen as a mix between the non-isothermal and the isothermal crystallization from the melt. The non-isothermal crystallization indeed corresponds to the stage where the molten material is injected into the mold, where a rapid cooling from the melt temperature to the mold temperature occurs. Then, the injected material follows an isothermal step, remaining at a constant temperature for the cooling time of the cycle, before its extraction.

**Figure 2:**
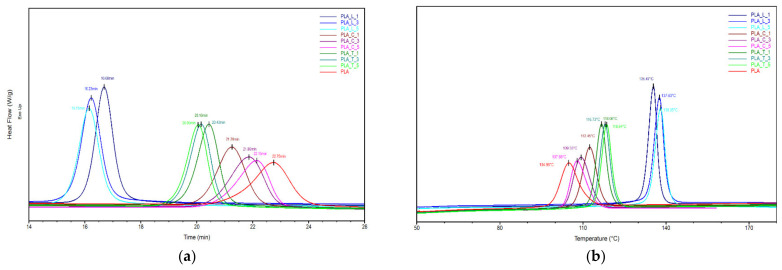
Non-isothermal crystallization curves from the melt showing the shift of the crystallization peak depending on the type of NA adopted, as a function of (**a**) time and (**b**) temperature.

**Figure 3:**
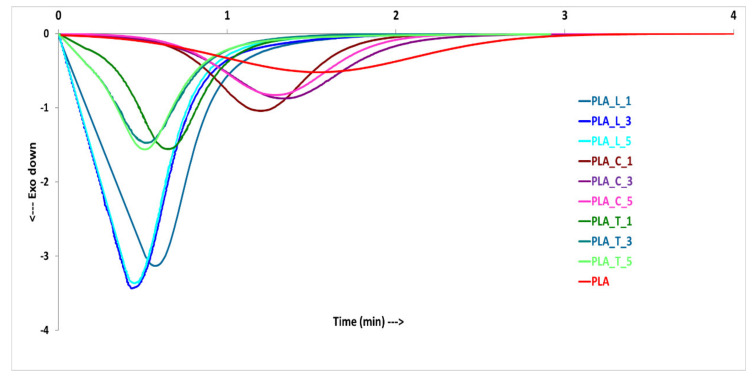
Isothermal crystallization traces at 110 °C.

The non-isothermal crystallization curves from the melt, shown in Figure 2, also compared to the isothermal curves of the mold temperature (110 °C) (Figure 3), are very useful to select the optimum nucleating agent content in order to produce injected molded specimens with high crystallinity content. 

The non-isothermal DSC cooling curves (Figure 2) show a great shift of the crystallization peak depending on the type of NAs adopted. LAK appears the most efficient, confirming the positive results found in literature [10,14,32], with the crystallization peak that appears initially (around 16 min) if compared to the other NAs. Despite the marked crystallization peak shift versus longer times (around 20 min), talc also works well as NA for PLA. Calcium carbonate seems less efficient with a further shift of the crystallization peak versus longer times (21–23 min). As it can be expected, neat PLA has the lowest crystallization peak temperature and the highest time, confirming the necessity of adding NAs to obtain—from a practical point of view—injection-molded samples with high crystallinity content and feasible injection molding cycle time. 

In Figure 3, the DSC traces recorded in isothermal melt crystallization at the mold temperature (110 °C) are reported. The obtained curves confirm that neat PLA has the slowest crystallization kinetic with a very broad crystallization peak. With the addition of NAs, the crystallization peak sharpens and the half crystallization time shortens, indicating that all the NAs are able to enhance PLA crystallization.

**Figure 4:**
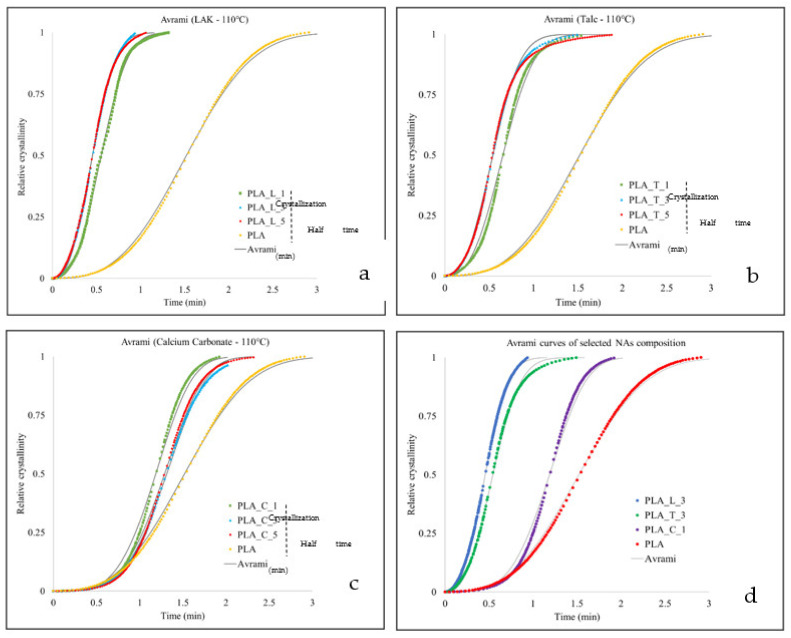
Variation of relative crystallinity with crystallization time at 110 °C for (**a**) LAK, (**b**) talc, (**c**) calcium carbonate; and (**d**) Avrami curves of selected NA compositions. The solid lines are the fitting curves according to the Avrami model.

Isothermal traces were used to investigate the isothermal melt crystallization kinetics by the above-reported Avrami equation (Section 2.3.2) and the plots of the relative crystallinity versus time are reported in Figure 4, where a good fitting between the theoretical and experimental values can be observed. From a practical point of view, it is useful to know the crystallization half time, that is the time taken by a sample to reach 50% of relative crystallinity. The crystallization half times, reported in Figure 4, show that LAK and talc at 3 wt % can be chosen for the subsequent injection molding process, because they have a crystallization half time equal to their counterparts containing 5 wt % of NAs. Calcium carbonate formulations are confirmed to be less efficient, having crystallization half times more than 1 min; however, PLA_C_1 is confirmed to be more efficient when compared to PLA_C_3 and PLA_C_5. In Figure 4d, the comparison between the Avrami curves of the best NA compositions is reported. It can be observed that PLA_L_3 and PLA_T_3 are comparable in isothermal crystallization (crystallization half times of 0.45 and 0.54 min, respectively) while they are different in non-isothermal crystallization (Figure 2). PLA_C_1 slightly improves the crystallization kinetics if compared to pure PLA (crystallization half time 1.19 min for PLA_C_1 and 1.52 min for PLA) but is not so effective as talc and LAK.

**Figure 5:**
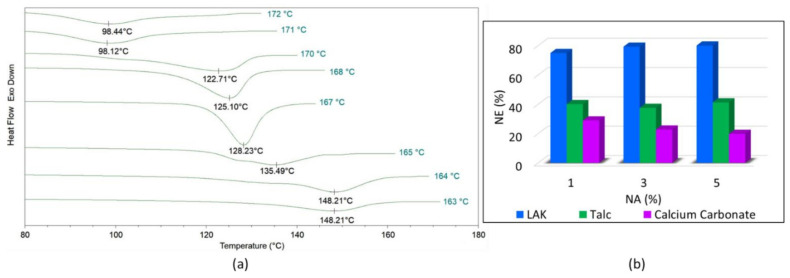
(**a**) DSC crystallization curves of neat PLA after self-nucleation at different self-nucleation temperatures. The T_c_ peak values are shown below each curve. (**b**) Nucleation efficiency (NE) of the various NAs.

To quantify the impact of the NAs addition on the PLA crystallinity, the nucleation efficiency (NE) of the various NAs was calculated by using Equation (4). *T*_*c*1_ and *T*_*c*2*,max*_ for PLA were determined and they were found equal to 98 °C and 148 °C respectively, as shown in Figure 5a. The upper and lower bound temperatures (*T*_*c*1_ and *T_C2,max_*) found are in good agreement with values reported in literature [32,48,51,52] with slight differences ascribable to the different PLA grades (different molecular weight, optical purity, as well as crystallization temperature). Using the *T_c_* obtained from non-isothermal crystallization upon cooling at 5 °C/min (Figure 2a), the NE of the various NAs was calculated and reported in Figure 5b. The NE results are coherent to what was observed from the isothermal and non-isothermal characterizations. It can be affirmed that LAK is very efficient, allowing PLA to reach a desired level of crystallinity within a short period of time (which is fundamental to maintaining short cycle times during injection molding). For the formulations containing LAK, the nucleation efficiency is very high (greater than 70%) for all compositions; however, considering the data of non-isotherms and Avrami curves, PLA_L_3 is the most promising because it has values very near to PLA_L_5 (the best) but with a lower LAK content (LAK is not commercially cheap). A similar choice was made for talc which is less efficient if compared to LAK, but its trend with concentration is very similar with little variations between PLA_T_3 and PLA_T_5; thus, PLA_T_3 was chosen for the successive steps.

Finally, calcium carbonate is confirmed to be the less efficient with a trend that it is negatively influenced by its concentration; the NE dramatically decreases with the CaCO_3_ content; for this reason, the composition with the lowest calcium carbonate content was selected (PLA_C_1) for the successive steps.

### 3.2. Injection Molded Specimen Results

The thermal properties of the injection molded samples referred to two different injection molding cycle times adopted (30 s and 1 min) are reported in Table 3. The first heating scan, as also reported in literature [10,12,14], best reflects the effect of the two different cycle times adopted for the evaluation of the final crystallinity, achieved by the injection molded samples.

**Figure 6:**
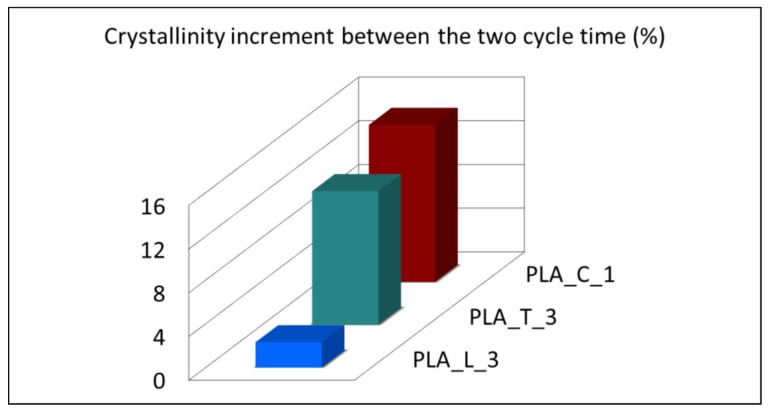
Crystallinity increment between the two cycle time adopted (30 s and 60 s) of the injection molded formulation containing different NAs.

Neat PLA exhibits its characteristic thermal properties [53]: the crystallinity content achieved is not very high—approximately 20%—with no ascribable differences among the two cycle times adopted, confirming the slow crystallization tendency of neat PLA to crystallize in absence of any NAs. No relevant *T_g_* variations were recorded, adding the different NA typologies. In agreement with literature [10,27], the addition of NAs shifts the cold crystallization peak and the melting peak toward lower and higher temperatures respectively. The absence of T_cc_ peak for PLA_L_3 is a good indication that LAK—at high molding temperatures—acts as an effective nucleating agent in promoting the PLA crystallization compared to other NAs adopted. It can be observed that, with LAK, no ascribable differences in the final crystallinity achieved are obtained among the two cycle times; this interesting aspect enables the adoption of lower cycle times, boosting injection molding productivity. On the other hand, talc is very sensitive to cycle time and it is comparable to LAK (with the T_cc_ disappearance) when a longer cycle time (60 s) is adopted. The poor calcium carbonate efficiency is confirmed, showing the lowest T_cc_ peak shift and the lowest crystallinity percentage value when compared to other NAs; calcium carbonate is also very sensitive to the cycle time and a marked improvement (highlighted in Figure 6) of the final crystallinity was achieved, adopting a cycle time of 60 s. 

The results of the mechanical and HDT tests are summarized in Table 4. As it can be expected, all formulations exhibit brittle failure, no yielding, and relatively high stress at break and low elongation at break values were registered. Increasing the crystallinity percentage, a further increment of the PLA stiffness has been registered, which causes an additional decrement of the elongation at break. In agreement with what is reported in the literature [10,22], the Young’s modulus increases with the crystallinity content; this behavior is ascribable to the increment of the PLA crystallinity fraction having a higher elastic modulus with respect to its amorphous counterpart [12]. The tensile stress shows very slight differences (most of them within the standard deviation); however, a slight decrement of tensile stress with the increment of crystallinity content can be noticed. This tensile stress behavior was found in a previous study [12], and it was correlated to the crystalline regions that act as stress concentrators leading to premature failure of the materials.

Impact strength and HDT are reported to be two important deciding factors concerning PLA commercialization in wide-scale applications [10]. The effect of these two properties correlated to the processing conditions (in particular to the injection cycle time) and the typology of NAs adopted is therefore considered. From the impact strength point of view, it can be observed a C.I.S. decrement correlated to the brittle crystalline fraction; for all formulations, the cycle time increment from 30 s to 60 s led to an increase in the crystalline fraction, with a consequent reduction in impact resistance. Nevertheless, it is interesting is to observe how C.I.S. is also strictly correlated to the typology of the NA adopted. In fact, LAK that is an organic NA is the most efficient from the point of view of the PLA crystallization ability (as observed in Section 3.1), but it does not mitigate the PLA embrittlement generated by the increment of the crystalline fraction and this behavior brings C.I.S. values below the net PLA. On the other hand, talc and calcium carbonate are inorganic fillers that can be used not only as NAs, but they also have been reported to increase the toughness of PLA-based systems [54]. Consequently, although less efficient than LAK, talc and calcium carbonate are able to counterbalance the detrimental effect on C.I.S. caused by the crystallinity increment, leading to C.I.S. values that are comparable or better when compared to pure PLA.

**Figure 7:**
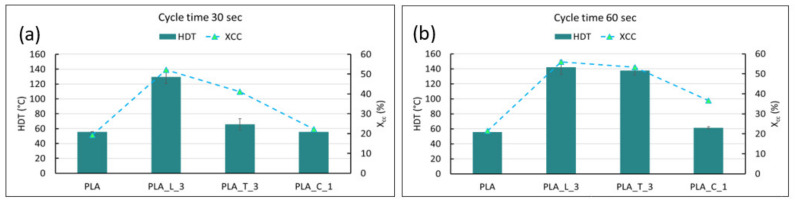
Crystallinity and HDT trends of the injection molded formulations for a cycle time of (**a**) 30 s and (**b**) 60 s.

With respect to HDT, this thermo-mechanical value reflects NAs’ efficiencies and crystallization kinetics; in fact, for LAK (which the most efficient in nucleating PLA, independently from the cycle time adopted) the highest HDT values can be observed (>120 °C). Talc, being more sensitive to the cycle time in crystallizing PLA, reaches high HDT values only when a cycle time of 60 s is adopted. Finally, calcium carbonate is also sensitive to the cycle time adopted, but with a low NE, it does not significantly increase the HDT, even if a cycle time of 60 s is adopted. It is interesting to observe how the increment of HDT is not proportionally dependent on the increase in crystallinity. It can be observed from Figure 7 that a crystallinity threshold for achievement improvements in HDT is necessary, similarly to what was observed by Tang et al. [49]. The crystallinity threshold to be achieved in order to significantly increase (>110°) the PLA HDT was found to be approximately equal to 50%. 

**Figure 8:**
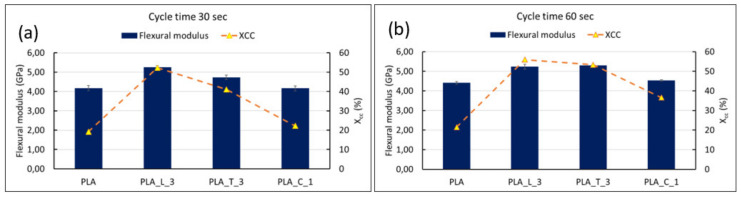
Crystallinity and flexural modulus trends of the injection molded formulations for a cycle time of (**a**) 30 s and (**b**) 60 s.

The flexural modulus is also influenced by the crystallinity percentage and its comparison with the crystallinity percentages of the samples (Figure 8) are similar to those of Figure 7. These results could be expected, as the HDT test is a 3PBD test in temperature mode. However, despite of HDT—in which a crystallinity threshold was observable for improving the HDT values—a strong dependence between the crystallinity content and the flexural modulus increment can be observed (Figure 8). The flexural modulus enhancement, differently from C.I.S., is not influenced by the NA typology. In fact, as also reported by Harris et al. [27], at this low loading level, the contribution to the flexural modulus from particle typology is negligible and it depends only by the crystallinity content. This is confirmed by the results achieved: LAK has the highest crystallinity content and the highest flexural modulus, while calcium carbonate—due to its low NE efficiency and low quantity added—does not significantly improve the PLA flexural modulus.

**Figure 9:**
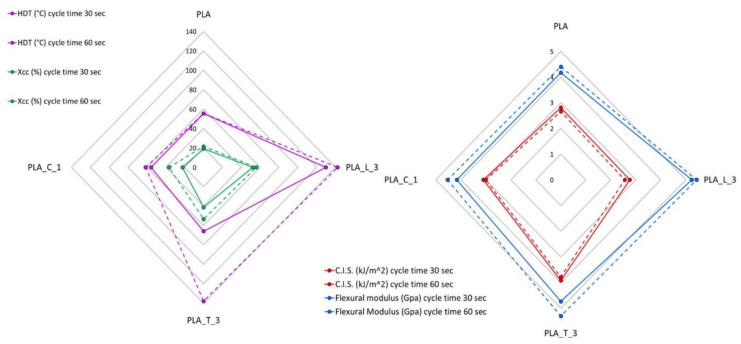
Comparison of the HDT, crystallinity, C.I.S. and Flexural modulus of the injection molded formulation for both cycle time adopted.

Comparing the overall properties mentioned above and correlating them to the cycle time adopted (Figure 9), it is possible to notice that the choice of the nucleating agent is not univocal. Rather, it depends strongly on the properties that must be improved and—especially from an industrial point of view—on the productivity/final costs of the final material. If the minimum cycle time is necessary and a high crystallinity value and HDT are also the final goal, LAK is the best choice. On the other hand, if the impact strength of PLA has to be maintained and an increment in flexural modulus is also required, together with a high HDT value, talc is the best choice—but, the cycle time has to be higher (60 s).

## 4. Conclusions

Injection molding parameters, such as mold temperature and cycle time, can affect productivity and energy consumption of the process, as well as the quality, thermo-mechanical performance, and cost of the final molded product. In fact, biopolymers such as PLA—if compared to conventional fossil-based polymers—need higher a mold temperature and longer cycle time to allow crystallization and to obtain improved HDT, stiffness, and chemical resistance. Cycle time is a crucial parameter to reduce the final material cost and to the widespread potential of PLA applications.

In this work, the mechanical and thermomechanical behavior of injection-molded PLA samples containing different commercial NAs (LAK, talc, and calcium carbonate) was investigated. The first objective was the selection of the best content of NAs in the PLA matrix that was able to achieve adequate crystallinity content in the shortest time. Carrying out a preliminary thermal characterization of the blends, the nucleation efficiency and isothermal and non-isothermal crystallization were calculated, and the best amounts of each NA were selected. As a consequence, for the injection molding process, two cycle times that can be industrially adopted (30 s and 60 s) were chosen and the samples were produced with the mold temperature set at 110 °C. Thus, the study focused on the effect of the injection molding cycle time on thermal and mechanical blends’ properties.

From the study, it emerged that the choice of the suitable nucleating agent is not univocal. LAK is the most efficient NA, and when it is added to PLA, it highly increases the PLA crystallinity (reaching high HDT values) regardless of the cycle time adopted; however, with LAK, decrements of flexural modulus and Charpy impact resistance of PLA have been registered. On the other hand, talc and calcium carbonate increase the flexural modulus and impact strength; however, calcium carbonate is not efficient in promoting PLA crystallization, while talc is efficient (high HDT value obtained) only if a cycle time of 60 s is adopted.

It can be concluded that it is possible to choose the nucleating agent to be added into PLA on the basis of the PLA characteristics to be improved, and based on the needs of the production (selecting the suitable injection molding cycle).

## Figures and Tables

**Table 1:** Formulation names and compositions.

Name	PLA (wt %)	LAK (wt %)	Talc (wt %)	CaCO_3_ (wt %)
PLA	100	-	-	-
PLA_L_1	99	1	-	-
PLA_L_3	97	3	-	-
PLA_L_5	95	5	-	-
PLA_T_1	99	-	1	-
PLA_T_3	97	-	3	-
PLA_T_5	95	-	5	-
PLA_C_1	99	-	-	1
PLA_C_3	97	-	-	3
PLA_C_5	95	-	-	5

**Table 2:** Injection molding conditions.

Main Injection Molding Parameters	
Temperature profile (°C)	180/185/190/190
Mold temperature (°C)	110
Injection and holding time (s)	5
Injection pressure (bar)	70
Cooling time (s)	25/55

**Table 3:** Results of the DSC first heating scan on injection molded specimens.

Blend Name	Cycle time (s)	Tg (°C)	Tcc (°C)	Tm (°C)	ΔHcc (J/g)	ΔHm (J/g)	Xcc (%)
PLA	30	58.6	98.6	176.4	29.6	47.5	19.2
PLA	60	58.5	97.9	176.9	23.9	44	21.5
PLA_L_3	30	58.7	-	177.7	-	47.1	52.2
PLA_L_3	60	58.8	-	177.7	-	50.4	55.9
PLA_T_3	30	58.9	90	177.4	13.3	50.3	41
PLA_T_3	60	58.5	-	177.7	-	48.1	53.3
PLA_C_1	30	58.8	93.8	176.8	28.6	49	22.2
PLA_C_1	60	58.6	91.4	177	14.7	48.4	36.6

**Table 4:** Tensile, flexural, C.I.S., and HDT results of the injection molded specimens.

Blend Name	Cycle Time (s)	Young Modulus (GPa)	Tensile Stress (MPa)	Elongation at Break (%)	Flexural Modulus (GPa)	Charpy Impact Strength C.I.S. (kJ/m^2^)	HDT (°C)
PLA	30	3.45 ± 0.09	59.20 ± 0.72	3.22 ± 0.44	4.17 ± 0.12	2.81 ± 0.28	55.4 ± 0.5
PLA	60	3.53 ± 0.03	60.30 ± 0.43	2.65 ± 0.10	4.40 ± 0.07	2.66 ± 0.13	55.5 ± 0.6
PLA_L_3	30	4.38 ± 0.01	53.07 ± 0.40	1.88 ± 0.07	5.24 ± 0.08	2.76 ± 0.25	129.7 ± 1.3
PLA_L_3	60	4.35 ± 0.03	54.32 ± 0.46	1.89 ± 0.05	5.43 ±0.12	2.56 ± 0.36	142.0 ± 1.1
PLA_T_3	30	4.23 ± 0.09	58.32 ± 0.94	2.33 ± 0.31	4.73 ± 0.11	3.92 ± 0.65	65.7 ± 0.9
PLA_T_3	60	4.52 ± 0.05	58.6 ± 0.41	1.85 ± 0.11	5.3 ± 0.06	3.78 ± 0.19	137.6 ± 1.1
PLA_C_1	30	3.51 ± 0.09	58.92 ± 0.53	2.72 ± 0.18	4.16 ± 0.13	3.10 ± 0.62	55.7 ± 0.3
PLA_C_1	60	3.62 ± 0.07	59.96 ± 0.65	2.46 ± 0.12	4.53 ± 0.03	3.00 ± 0.65	61.4 ± 1.5

## Data Availability

The study did not report any data.

## References

[1] Okada M. (2002). Chemical syntheses of biodegradable polymers. Prog. Polym. Sci..

[2] Cheng Y., Deng S., Chen P., Ruan R. (2009). Polylactic acid (PLA) synthesis and modifications: A review. Front. Chem. China.

[3] Pietrosanto A., Scarfato P., Di Maio L., Nobile M.R., Incarnato L. (2020). Evaluation of the Suitability of Poly(Lactide)/Poly(Butylene-Adipate-co-Terephthalate) Blown Films fro Chilled and Frozen Food Packaging Applications. Polymers.

[4] Narancic T., Cerrone F., Beagan N. (2020). Recent Advances in Bioplastics: Application and Biodegradation. Polymers.

[5] Rusu D., Boyer S., Lacrampe M.F., Krawczak P. (2011). Bioplastics for automotive applications. Handbook of Bioplastics and Biocomposites Engineering Applications.

[6] Coltelli M.-B., Gigante V., Cinelli P., Vannozzi A., Aliotta L., Lazzeri A. (2021). Biobased and biodegradable rigid and flexible polymeric packaging. An Introduction to the Circular Economy.

[7] Ray S.S., Bousmina M. (2005). Biodegradable polymers and their layered silicate nanocomposites: In greening the 21st century materials world. Prog. Mater. Sci..

[8] Gruber P.R., Drumright R.E., Henton D.E. (2000). Polylactic acid technology. Adv. Mater.

[9] Auras R., Harte B., Selke S. (2004). An overview of polylactides as packaging materials. Macromol. Biosci..

[10] Nagarajan V., Zhang K., Misra M., Mohanty A.K. (2015). Overcoming the fundamental challenges in improving the impact strength and crystallinity of PLA biocomposites: Influence of nucleating agent and mold temperature. ACS Appl. Mater. Interfaces.

[11] Li H., Huneault M.A. (2007). Effect of nucleation and plasticization on the crystallization of poly(lactic acid). Polymer.

[12] Aliotta L., Gazzano M., Lazzeri A., Righetti M.C. (2020). Constrained Amorphous Interphase in Poly (L -lactic acid): Estimation of the Tensile Elastic Modulus. ACS Omega.

[13] Cocca M., Di Lorenzo M.L., Malinconico M., Frezza V. (2011). Influence of crystal polymorphism on mechanical and barrier properties of poly(l-lactic acid). Eur. Polym. J..

[14] Aliotta L., Cinelli P., Coltelli M.B., Righetti M.C., Gazzano M., Lazzeri A. (2017). Effect of nucleating agents on crystallinity and properties of poly (lactic acid) (PLA). Eur. Polym. J..

[15] Pan P., Inoue Y. (2009). Polymorphism and isomorphism in biodegradable polyesters. Prog. Polym. Sci..

[16] Kawai T., Rahman N., Matsuba G., Nishida K., Kanaya T., Nakano M., Okamoto H., Kawada J., Usuki A., Honma N. (2007). Crystallization and melting behavior of poly (L-lactic acid). Macromolecules.

[17] Androsch R., Schick C., Lorenzo M.L. (2014). Di Melting of Conformationally Disordered Crystals (α′-Phase) of Poly(l-lactic acid). Macromol. Chem. Phys..

[18] Zhang J., Tashiro K., Tsuji H., Domb A.J. (2008). Disorder-to-order phase transition and multiple melting behavior of poly(L-lactide) investigated by simultaneous measurements of WAXD and DSC. Macromolecules.

[19] Pan P., Zhu B., Kai W., Dong T., Inoue Y. (2008). Effect of crystallization temperature on crystal modifications and crystallization kinetics of poly (L-lactide). J. Appl. Polym. Sci..

[20] Navrátilová N., Náplava A. (2011). Study of Biodegradable Plastics Produced By Injection Molding. Mater. Sci. Technol..

[21] Dang X.P. (2014). General frameworks for optimization of plastic injection molding process parameters. Simul. Model. Pract. Theory.

[22] Suryanegara L., Okumura H., Nakagaito A.N., Yano H. (2011). The synergetic effect of phenylphosphonic acid zinc and microfibrillated cellulose on the injection molding cycle time of PLA composites. Cellulose.

[23] Kfoury G., Raquez J.-M., Hassouna F., Odent J., Toniazzo V., Ruch D., Dubois P. (2013). Recent advances in high performance poly (lactide): From “green” plasticization to super-tough materials via (reactive) compounding. Front. Chem..

[24] Mathew A.P., Oksman K., Sain M. (2006). The effect of morphology and chemical characteristics of cellulose reinforcements on the crystallinity of polylactic acid. J. Appl. Polym. Sci..

[25] Liu H., Zhang J. (2011). Research progress in toughening modification of poly(lactic acid). J. Polym. Sci. Part B Polym. Phys..

[26] Ljungberg N., Wesslen B. (2002). The effects of plasticizers on the dynamic mechanical and thermal properties of poly (lactic acid). J. Appl. Polym. Sci..

[27] Harris A.M., Lee E.C. (2008). Improving mechanical performance of injection molded PLA by controlling crystallinity. J. Appl. Polym. Sci..

[28] Kawamoto N., Sakai A., Horikoshi T., Urushihara T., Tobita E. (2007). Nucleating agent for poly (l-lactic acid)—An optimization of chemical structure of hydrazide compound for advanced nucleation ability. J. Appl. Polym. Sci..

[29] Tsuji H., Takai H., Saha S.K. (2006). Isothermal and non-isothermal crystallization behavior of poly (L-lactic acid): Effects of stereocomplex as nucleating agent. Polymer.

[30] Nam J.Y., Okamoto M., Okamoto H., Nakano M., Usuki A., Matsuda M. (2006). Morphology and crystallization kinetics in a mixture of low-molecular weight aliphatic amide and polylactide. Polymer.

[31] Lim L.T., Auras R., Rubino M. (2008). Processing technologies for poly(lactic acid). Prog. Polym. Sci..

[32] Feng Y., Ma P., Xu P., Wang R., Dong W., Chen M., Joziasse C. (2018). The crystallization behavior of poly(lactic acid) with different types of nucleating agents. Int. J. Biol. Macromol..

[33] Aliotta L., Gigante V., Coltelli M., Cinelli P., Lazzeri A., Seggiani M. (2019). Thermo-Mechanical Properties of PLA/Short Flax Fiber Biocomposites. Appl. Sci..

[34] Wang Y., Tong B., Hou S., Li M., Shen C. (2011). Transcrystallization behavior at the poly (lactic acid)/sisal fiber biocomposite interface. Compos. Part A Appl. Sci. Manuf..

[35] Wang L., Wang Y., Huang Z., Weng Y. (2015). Heat resistance, crystallization behavior, and mechanical properties of polylactide/nucleating agent composites. Mater. Des..

[36] Schäfer H., Pretschuh C., Brüggemann O. (2019). Reduction of cycle times in injection molding of PLA through bio-based nucleating agents. Eur. Polym. J..

[37] Ageyeva T., Kovács J.G., Tábi T. (2021). Comparison of the efficiency of the most effective heterogeneous nucleating agents for Poly(lactic acid). J. Therm. Anal. Calorim..

[38] Petchwattana N., Narupai B. (2019). Synergistic Effect of Talc and Titanium Dioxide on Poly(lactic acid) Crystallization: An Investigation on the Injection Molding Cycle Time Reduction. J. Polym. Environ..

[39] Wei Z., Shao S., Sui M., Song P., He M., Xu Q., Leng X., Wang Y., Li Y. (2019). Development of zinc salts of amino acids as a new class of biocompatible nucleating agents for poly(L-lactide). Eur. Polym. J..

[40] Gong X., Pan L., Tang C.Y., Chen L., Li C., Wu C., Law W.C., Wang X., Tsui C.P., Xie X. (2016). Investigating the crystallization behavior of poly(lactic acid) using CdSe/ZnS quantum dots as heterogeneous nucleating agents. Compos. Part B Eng..

[41] Tang H., Chen J.-B., Wang Y., Xu J.Z., Hsiao B.S., Zhong G.J., Li Z.M. (2012). Shear flow and carbon nanotubes synergistically induced nonisothermal crystallization of poly(lactic acid) and its application in injection molding. Biomacromolecules.

[42] Avrami M. (1940). Kinetics of phase change. II transformation-time relations for random distribution of nuclei. J. Chem. Phys..

[43] Wang Y., He D., Wang X., Cao W., Li Q., Shen C. (2013). Crystallization of poly(lactic acid) enhanced by phthalhydrazide as nucleating agent. Polym. Bull..

[44] He D., Wang Y., Shao C., Zheng G., Li Q., Shen C. (2013). Effect of phthalimide as an efficient nucleating agent on the crystallization kinetics of poly(lactic acid). Polym. Test..

[45] Fischer E.W., Sterzel H.J., Wegner G. (1973). Investigation of the structure of solution grown crystals of lactide copolymers by means of chemical reactions. Kolloid-Z. Z. Polym..

[46] Fillon B., Thierry A., Lotz B., Wittmann J.C. (1994). Efficiency scale for polymer nucleating agents. J. Therm. Anal..

[47] Fillon B., Wittmann J.C., Lotz B., Thierry A. (1993). Self-nucleation and recrystallization of isotactic polypropylene (α phase) investigated by differential scanning calorimetry. J. Polym. Sci. Part B Polym. Phys..

[48] Schmidt S.C., Hillmyer M.A. (2001). Polylactide Stereocomplex Crystallites as Nucleating Agents for Isotactic Polylactide. J. Polym. Sci. Part B Polym. Phys..

[49] Tang Z., Zhang C., Liu X., Zhu J. (2012). The crystallization behavior and mechanical properties of polylactic acid in the presence of a crystal nucleating agent. J. Appl. Polym. Sci..

[50] Aliotta L., Vannozzi A., Canesi I., Cinelli P., Coltelli M., Lazzeri A. (2021). Poly(lactic acid) (PLA)/Poly(butylene succinate-co-adipate) (PBSA) Compatibilized Binary Biobased Blends: Melt Fluidity, Morphological, Thermo-Mechanical and Micromechanical Analysis. Polymers.

[51] Jalali A., Huneault M.A., Elkoun S. (2017). Effect of molecular weight on the nucleation efficiency of poly(lactic acid) crystalline phases. J. Polym. Res..

[52] Kovalcik A., Pérez-Camargo R.A., Fürst C., Kucharczyk P., Müller A.J. (2017). Nucleating efficiency and thermal stability of industrial non-purified lignins and ultrafine talc in poly(lactic acid) (PLA). Polym. Degrad. Stab..

[53] Pyda M., Bopp R.C., Wunderlich B. (2004). Heat capacity of poly (lactic acid). J. Chem. Thermodyn..

[54] Aliotta L., Cinelli P., Beatrice Coltelli M., Lazzeri A. (2018). Rigid filler toughening in PLA-Calcium Carbonate composites: Effect of particle surface treatment and matrix plasticization. Eur. Polym. J..

